# A novel approach to screen and compare emission inventories

**DOI:** 10.1007/s11869-016-0402-7

**Published:** 2016-04-12

**Authors:** P. Thunis, B. Degraeuwe, K. Cuvelier, M. Guevara, L. Tarrason, A. Clappier

**Affiliations:** European Commission, Institute for Environment and Sustainability, Ispra, Italy; Ex-European Commission, Institute for Environment and Sustainability, Ispra, Italy; Barcelona Supercomputing Center, Earth Sciences Division, Barcelona, Spain; Norwegian Institute for Air Research, Urban Environment and Industry, Lillestroem, Norway; Laboratoire Image Ville Environnement, Université de Strasbourg, Strasbourg, France

**Keywords:** Emission inventories, Activity data, Emission factors

## Abstract

A methodology is proposed to support the evaluation and comparison of different types of emission inventories. The strengths and weaknesses of the methodology are presented and discussed based on an example. The approach results in a “diamond” diagram useful to flag out anomalous behaviors in the emission inventories and to get insight in possible explanations. In particular, the “diamond” diagram is shown to provide meaningful information in terms of: discrepancies between the total emissions reported by macro-sector and pollutant, contribution of each macro-sector to the total amount of emissions released by pollutant, and the identification and quantification of the different factors causing the discrepancies between total emissions. A practical example in Barcelona is used for testing and to provide relevant information for the analyzed emission datasets. The tests show the capability of the proposed methodology to flag inconsistencies in the existing inventories. The proposed methodology system may be useful for regional and urban inventory developers as an initial evaluation of the consistency of their inventories.

## Introduction

Emission inventories are essential in explaining observed variability and trends in atmospheric composition. They are generally identified as key inputs in the air quality modelling chain, especially when they are used to support regulatory decisions, such as for air quality planning or for the assessment of concentration levels over a given territory (EEA 2011; ETC and ACM 2013). At the same time, studies point out to emissions as the most uncertain factor among the different components of air quality models (e.g., meteorology, boundary conditions, model parameters) (Russell and Dennis 2000; Francois et al. 2005; Davison et al. 2011; Viaene et al. 2013). The development of accurate emission inventories is therefore particularly relevant to air quality applications because this will determine to a large extent the accuracy of the subsequent air quality models (Tong et al. 2011; Frost et al. 2013; Fann et al. 2009; De Fatima Andrade et al. 2012).

Building an emission inventory for a given urban area, region, or country is always a challenge as highly detailed local information for a large variety of emission sources needs to be collected. Such information is not always available with the requested level of accuracy for all emitting sectors and/or pollutants. Two methodological approaches for compiling emission inventories, often referred to as “top-down” and “bottom-up,” exist that both require information concerning the amount of activity “A” (e.g., fuel consumption, vehicle kilometers travelled) and emission factors associated to this activity “e” (e.g., amount of pollutant emitted per activity unit). Emissions are then estimated as the product of the emission factor with the relevant activity data. The main difference between “top-down” and “bottom-up” approaches relies on the specificity of the emission factor selected and the spatial and temporal data aggregation level in which the activity is collected. In the case of top-down approaches, activity data is first collected at national or regional level and then distributed over the grids of the modelling domain based on information or surrogate data that is representative of the activity (e.g., population density). In the case of a bottom-up approach, the activity data is collected on a fine spatial scale (e.g., facility level for industrial emissions or road level for traffic emissions) and aggregated to the required spatial resolution of the air quality model. It is common to use both top-down and bottom-up methodologies to develop a single emission inventory. However, emission inventories at national level mostly rely on top-down approaches, while emission inventories developed for local and urban applications rely to a larger degree on bottom-up approaches. Given their diversity in terms of methodology, these two approaches often do not lead to comparable emission estimates. In this work, we propose a simple methodology to compare emission inventories and to identify the key factors on which acting to improve consistency.

The use of this methodology must be seen as a first step in an emission inter-comparison exercise to be completed with further evaluation and comparison indicators (such as introduced in the FAIRMODE emission benchmarking tool) (Guevara et al. 2015) or with other approaches such as a GIS comparison exercise (i.e., spatial cross-checking between layers of input data and emission results) or a comparison of air quality model simulations using multiple emission datasets and observational data.

## Methodology: the “diamond” diagram

The main goal of the methodology is to allow for a better understanding of the reasons for discrepancies between emission estimates over a given area. The intention is to screen a bottom-up emission inventory and highlight possible sources of inconsistencies when compared with standardized existing top-down inventories. It must be noted however that the diamond approach may be applied to any type of inventories. The “diamond” diagram proposed here aims at identifying whether differences between inventories can be mostly related to differences: (1) in the use of emission factors or (2) in the choice of activity data. The methodology also delivers some quantitative assessment of the underlying uncertainty. It is important to note that no reference can be set in terms of emissions so that the resulting analysis remains relative, meaning that no information can be retrieved about the correctness of one inventory with respect to the other. Only differences can be highlighted.

We assume here to have two emission inventories over a given geographical area, detailed with similar nomenclatures in terms of emitted pollutants and sectors. If this is not the case, correspondences between the sectors should be built prior to the analyses. Note that this step is not specific to the approach proposed here but needs to be performed before any inventory inter-comparison. It is important, however, to stress that this step might lead to possible inconsistencies between emissions inventories that are rather artifacts than real mismatches. The necessary input data are the emission totals, detailed in terms of pollutant and activity sector (e.g., SNAP macro-sectors).

### Diamond diagram for a single source technology

For convenience, we start our derivation by considering a single technology before extending it to the more complex case of a macro-sector (set of technologies).

We consider emission estimates from two inventories (denoted below with the subscripts 1 and 2) for a given technology. Our aim is to express the emission factor ratio *e*_1_^*t*,*p*^/*e*_2_^*t*,*p*^ (for a technology *t* and a pollutant *p*) and the activity ratio *A*_1_^*t*^/*A*_2_^*t*^ from the only information we have, i.e., the two emission totals *E*_1_^*t*,*p*^ and *E*_2_^*t*,*p*^. Nor the emission factors neither the activities need to be known a priori.

From the relation linking total emissions to activity level and emission factors: *E*^*t*,*p*^ = *A*^*t*,*p*^*e*^*t*,*p*^, we can express the activity and emission factor ratios as follows:

1 $$ \frac{A_1^{t,p}}{A_2^{t,p}}=\frac{E_1^{t,p}}{E_2^{t,p}}\frac{e_2^{t,p}}{e_1^{t,p}} $$

2 $$ \frac{e_1^{t,p}}{e_2^{t,p}}=\frac{E_1^{t,p}}{E_2^{t,p}}\frac{A_2^{t,p}}{A_1^{t,p}} $$

To simplify these expressions and make them depend only on the known emission totals (E), we assume that, for a given technology, (t) one pollutant species (denoted as p*) can serve as a reference and satisfy the following two conditions:

3 $$ \forall t,\kern1em \exists p*\kern0.5em \mathrm{so}\ \mathrm{that}\ {\mathrm{e}}_1^{t,p*}={e}_2^{t,p*} $$

4 $$ \forall t,\forall p,\kern1em \frac{A_1^{t,p*}}{A_2^{t,p*}}=\frac{A_1^{t,p}}{A_2^{t,p}}=\frac{A_1^t}{A_2^t} $$

The first assumption (3) means that, for a given technology, we are confident that the emission factors for at least one pollutant are similar in the two inventories. For instance, the gasoline Euro-5 NO_*x*_ emission factor is supposed to be more certain than the primary PM_10_ emission factors for the same technology. The second assumption (4) is easier to fulfill since activities are generally independent of the pollutant (e.g., Amann et al. 2011). For the example of the traffic sector, this implies that the same number of cars is responsible for the emissions of the different pollutants. In our case, the assumption is even less stringent because we only require that the activity ratios are independent of the emitted pollutants.

With these assumptions, eq. (1) can be written for p* as:

5 $$ \frac{A_1^{t,p*}}{A_2^{t,p*}}=\frac{E_1^{t,p*}}{E_2^{t,p*}}\frac{e_2^{t,p*}}{e_1^{t,p*}}=\frac{E_1^{t,p*}}{E_2^{t,p*}}=\frac{A_1^t}{A_2^t} $$

where the last equality results from the assumption that the activity ratio is independent of the emitted pollutant. Using Eqs. (5) into (2) leads to:

6 $$ \frac{e_1^{t,p}}{e_2^{t,p}}=\frac{E_1^{t,p}}{E_2^{t,p}}\frac{E_2^{t,p*}}{E_1^{t,p*}} $$

It is important to note that the ratios given by (5) and (6) are now expressed only in terms of the total emissions.

We can then express the total emissions (E) in terms of the previous activity and emission factor ratios through the following equality:

7 $$ \log \left(\frac{E_1^{t,p}}{E_2^{t,p}}\right)= \log \left(\frac{A_1^t{e}_1^{t,p}}{A_2^t{e}_2^{t,p}}\right)= \log \left(\frac{A_1^t}{A_2^t}\right)+ \log \left(\frac{e_1^{t,p}}{e_2^{t,p}}\right) $$

Note that this equation is independent from the assumption made on the reference pollutant p*.

**Fig. 1:**
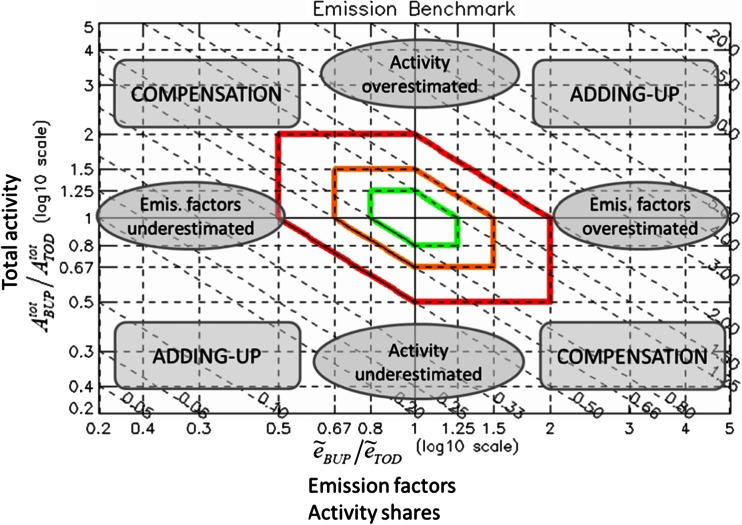
Diamond diagram. The X- and Y-axes indicate the discrepancies between the two inventories (BUP and TOD) in terms of emission factor and activity rate, respectively. The diagonal ratio iso-lines are indicative of discrepancies in terms of total emissions. The *colored* iso-lines delimitate the areas where the three factors: emission totals, activity rate, and emission factors are all within a given threshold

Ratios (5) and (6) are used to construct the “diamond” diagram which enables comparing the two inventories in terms of emission factors and activity levels (Fig. 1). The X-axis represents the emission factor ratio in a logarithmic scale log(*e*_1_/*e*_2_) while the Y-axis represents the activity data ratio in a logarithmic scale log(*A*_1_/*A*_2_). Relation (7) is then used to construct emission ratio iso-lines. The choice of the logarithmic scale allows visualizing relatively large differences which may occur between two inventories for given sectors or pollutants and results in a symmetric diagram for both over and under reporting. The distance from the X and Y origin provides information on the deviation (i.e., under- and over-prediction) made in terms of emission factor and activity, respectively. The emission ratio iso-lines (lines with slope −1) provide information on the overall under/over prediction in terms of emission totals. Colored thick lines are overlaid to delimitate where activity, emission factor, and total emissions all remain within a given degree of variation forming a diamond shape. The red diamond indicates ratios of activity, emission factor, and total emissions all within 100% (or a factor 2) differences. Compensation effects such as an over-prediction of emission factors linked to an under-prediction of the activity levels estimates are also identifiable on this diagram. These compensation points would lie on the diagonal but outside of the diamond areas.

The actual ratios between the inventories are then introduced as single elements in the “diamond” diagram. Different colors are used to identify each technology, and different symbols are used to distinguish pollutants. The size of the symbol is proportional to the emission magnitude. This feature is introduced to identify the biggest contributors and potential sectors that need attention. For example, a point far away from the origin but of small size could indicate a second order problem.

### Diamond diagram for a macro-sector with multiple technologies

The emissions corresponding to a macro-sector can be seen as a sum of emissions from the different technologies contributing to that macro-sector, i.e.,: ${E}^{m,p}={\displaystyle \sum_t{E}^{t,p}}={\displaystyle \sum_t{A}^{t,p}{e}^{t,p}}$ which we can re-write as:

8 $$ {E}^{m,p}={\displaystyle \sum_t{A}^{t,p}}\frac{{\displaystyle \sum_t{A}^{t,p}{e}^{t,p}}}{{\displaystyle \sum_t{A}^{t,p}}}={A}^{tot,m,p}\kern0.5em {\tilde{e}}^{m,p} $$

where *m* identifies the macro-sector that is source of the emissions, *A*^*tot*,*m*,*p*^ is the sum of all activities for pollutant “p” (from all technologies) within the macro-sector (e.g., kilometers driven for transport) and *ẽ*^*m*,*p*^ is an activity weighted emission factor for the macro-sector. This weighted macro-sector emission factor can be re-written as:

$$ {\tilde{e}}^{m,p}={\displaystyle \sum_t\frac{A^{t,p}}{A^{tot,m,p}}{e}^{t,p}}={\displaystyle \sum_t{a}^{t,m,p}{e}^{t,p}} $$

where the symbol *a* is used to denote the relative activity shares within a given macro-sector.

Similarly to the single technology derivation, we assume that, for each macro-sector, a reference pollutant can be identified, characterized by: (1) similar weighted emission factors and (2) activity ratios which do not depend on pollutant within that macro-sector. We can then generalize Eqs. (3) and (4) as:

$$ \begin{array}{l}\forall m,\kern1em \exists p*\ \mathrm{so}\ \mathrm{that}\kern0.75em {\tilde{e}}_1^{m,p*}={\tilde{e}}_2^{m,p*}\hfill \\ {}\forall m,\kern0.5em \forall p\kern1.5em \frac{A_1^{tot,m,p*}}{A_2^{tot,m,p*}}=\frac{A_1^{tot,m,p}}{A_2^{tot,m,p}}=\frac{A_1^{tot,m}}{A_2^{tot,m}}\hfill \end{array} $$

Relations (5) and (6) can then be generalized similarly as:

$$ \frac{A_1^{tot,m}}{A_2^{tot,m}}=\frac{E_1^{m,p*}}{E_2^{m,p*}} $$ and $$ \frac{{\tilde{e}}_1^{m,p}}{{\tilde{e}}_2^{m,p}}=\frac{E_1^{m,p}}{E_2^{m,p}}\frac{E_2^{m,p*}}{E_1^{m,p*}} $$

Similarly to the case of a single technology, Eq. (7) can be generalized to obtain the total emission ratio: *E*_1_^*m*,*p*^/*E*_2_^*m*,*p*^ for a macro-sector over the region of interest and derive the uncertainty iso-lines:

$$ \log \left(\frac{E_1^{m,p}}{E_2^{m,p}}\right)= \log \left(\frac{A_1^{tot,m}}{A_2^{tot,m}}\right)+ \log \left(\frac{{\tilde{e}}_1^{m,p}}{{\tilde{e}}_2^{m,p}}\right) $$

Since in this case the diamond diagram is based on aggregated quantities per macro-sector (i.e., weighted emission factors and total activity), it is necessary to take some precaution for its interpretation:

- The weighted emission factor (*ẽ*) includes activity shares in its definition. In the case of a single technology, the total emission is the product of two independent quantities: the emission factor and the activity. On the contrary, in the case of multiple technologies, Eq. (8) shows that the total emission is the product of two dependent quantities. Indeed, the weighted emission factors depend on activity shares. The differences seen in the diagram along the X-axis can therefore not be directly related to the single technology emission factors. A few examples are provided in the next subsection.
- Because the total activity (Y-axis) represents the sum of all technology activities, compensation effects (i.e., a positive activity change for one technology compensated by a negative change in another) will not be seen on the diagram.

It is important to note that an analysis at macro-sector level should be seen as a first step in the screening process, to identify the sectors/pollutant discrepancies. A more detailed approach in terms of sectors should then be used to identify the causes behind these differences.

### Interpretation of the “diamond” diagram

As mentioned, the interpretation of the X-axis of the diamond diagram is sometimes misleading due to the mix of information related to activities (activity share) and emission factor in the definition of *ẽ*.

Examples on how to interpret the diamond diagram are presented in this sub-section. We focus for simplicity on the case of a macro-sector composed of two different technologies (t_1_ and t_2_), each characterized by a specific emission factor. For a pollutant *p*, the two inventories (1 and 2) to be used in this comparison exercise can be expressed as follows:

$$ \begin{array}{l}{E}_1^{m,p}={A}_1^{t_1}{e}_1^{t_1,p}+{A}_1^{t_2}{e}_1^{t_2,p}={A}_1^{tot}\left({a}_1^{t_1}{e}_1^{t_1,p}+{a}_1^{t_2}{e}_1^{t_2,p}\right)={A}_1^{tot}{\tilde{e}}_1^{m,p}\\ {}{E}_2^{m,p}={A}_2^{t_1}{e}_2^{t_1,p}+{A}_2^{t_2}{e}_2^{t_2,p}={A}_2^{tot}\left({a}_2^{t_1}{e}_2^{t_1,p}+{a}_2^{t_2}{e}_2^{t_2,p}\right)={A}_2^{tot}{\tilde{e}}_2^{m,p}\end{array} $$

**Fig. 2:**
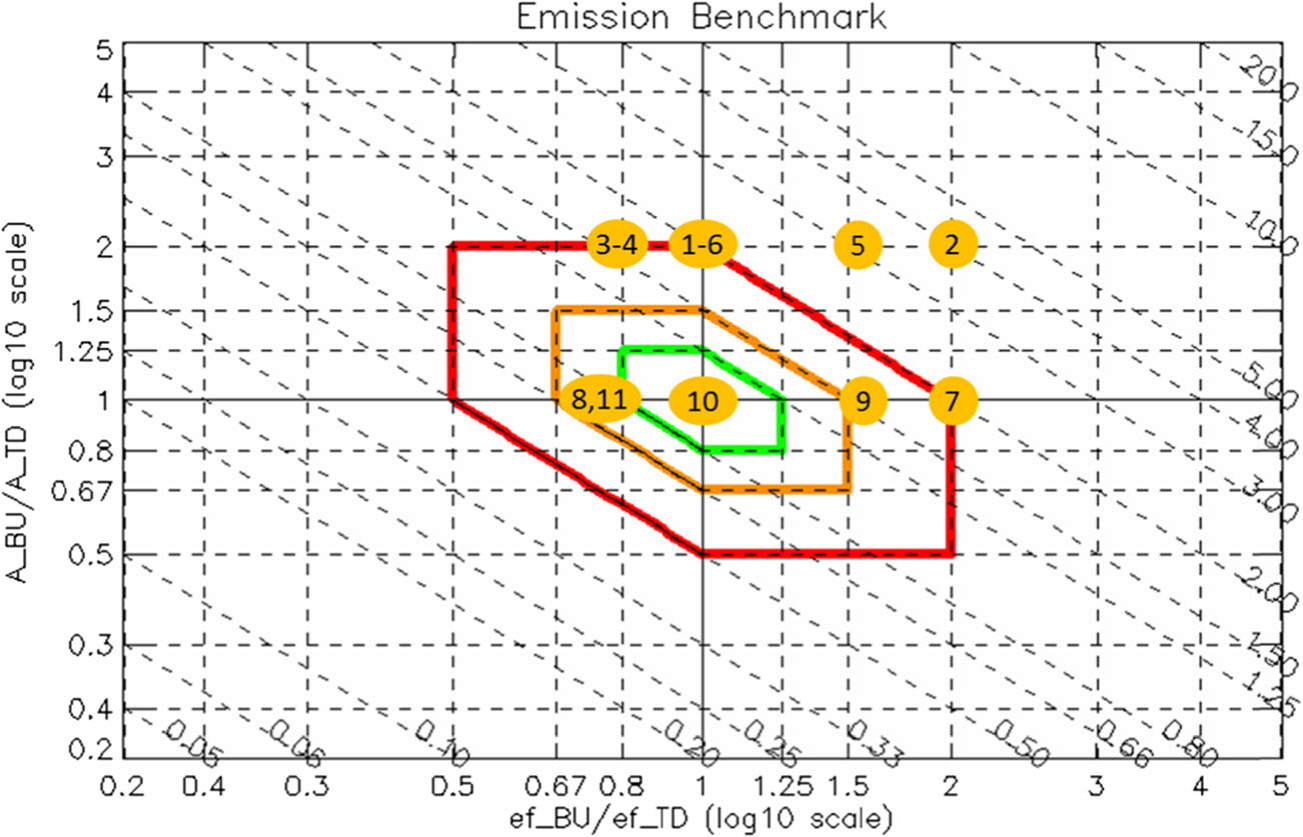
Diamond diagram highlighting the 11 points positions defined in Table 1

We will use the first inventory as reference and impose arbitrary variations to the second inventory in terms of total activity (*A*_tot_), activity shares (*a*), and emission factors (*e*). Table 1 provides an overview of the possible variations of these three factors, while Fig. 2 shows how these variations would be seen graphically. The left columns (orange) in Table 1 provide values for the reference inventory: the total activity (*A*_ref_), the activity shares (*a*_1_ and *a*_2_) and the two emission factors (*e*_1_ and *e*_2_). The weighted emission factor (*ẽ*_*ref*_) and total emissions (*E*_ref_) are calculated from these values. Note that these reference inventory values are kept unchanged for all the examples in Table 1. We then assume arbitrary variations of the total activity, activity shares, and emission factors (yellow columns) and calculate similarly the weighted emission factor (*ẽ*) and total emissions for the test inventory. From these values, the X and Y position in the diagram are obtained (green columns, Table 1) together with the ratio of the total emissions (Z column, Table 1) which informs about the overall under- or over estimation in terms of total emissions. The next three columns (in gray) summarize the changes in total activity (ΔA), activity shares (Δa1 Δa2), and emission factors (Δe1 Δe2) (↑ for an overestimation, ↔for a status quo and ↓ for an underestimation). The last column provides the reference number of the example used to identify the cases in Fig. 2. Note that only overestimation and compensation are tested here because underestimation would lead to similar interpretations.

**Table 1:**
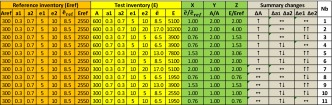
Comparison of two emission inventories with variations imposed in terms of three factors (total activity (A), activity shares (a), and emission factors (e))

The left most columns (*orange*) provide the values for the total activity, the two emission factors, and the activity shares for the reference inventory. From these values, the weighted emission factor (*ẽ*) and total emissions (E) are calculated. Arbitrary variations are assumed in the test inventory (*yellow columns*) and the weighted emission factor (*ẽ*) and total emissions are calculated similarly. From these values, the X and Y position in the diamond diagram are obtained (*green columns*) together with the ratio of the total emissions (Z column). The next three columns (in *gray*) summarize the changes in total activity (ΔA), activity shares (Δa1 Δa2), and emission factors (Δe1 Δe2) (↑ for an overestimation, ↔for a status quo and ↓ for an underestimation) between the two inventories. The last column provides the reference number of the scenario, used in Fig. 2

It is noteworthy to highlight the following points:

- Whatever the changes in terms of activity share and emission factors, the variations in terms of total activity (A_tot_) are always seen directly along the Y-axis. Indeed, the Y coordinates of points 7 to 11 are equal to 1 because their total activity are equal in both inventories, while the Y coordinates for points 1 to 6 are equal to 2, corresponding to an underestimation by a factor 2 of the total activity.
- Changes of both emission factors (e_1_ and e_2_) in similar ways (i.e., ↓↓ or ↑↑) (e.g., point 7) are directly visualized along the X-axis.
- Impacts of changing only the activity share (e.g., point 11) are seen on the X-axis because weighted emission factor include activity shares in their definition.
- Changes in activity compensated by changes in emission factors (e.g., point 10) cannot be identified.
- Changes in emission factors only (point 8) or in activity shares and emission factors that do not compensate each other (point 9) are reflected on the X-axis.
- The diamond diagram does not allow differentiating between:
  - Changes in emission factors compensated by changes in activity shares (point 8–11 or points 3–4)
  - Compensations in both activity shares and emission factors (points 1–6)

Due to the possible compensations, a position close to the origin does not guarantee that deviations between the two inventories are negligible, but positions far away from this origin are indicative of deviations.

The two main assumptions required to create the diamond diagram have the following implications:

- Assumption 1: A reference pollutant that uses the same emission factors per macro-sector in the two inventories can be selected. The points corresponding to the reference pollutant (p*) will consequently always lay along the vertical axis for all macro-sectors. Note that the choice of the reference pollutant is left to the user.
- Assumption 2: The activity level ratio per macro-sector between two inventories is the same for all pollutants. For a given macro-sector, the points corresponding to different pollutants will consequently always lay on the same horizontal line in the diagram.

## A case study: Barcelona

This section presents an example of the use of the diamond diagram to compare bottom-up and top-down emission inventories. We compare a Barcelona City local/regional inventory including a large amount of “bottom-up” information (i.e., HERMESv2.0; Guevara et al. 2013; hereinafter referred to as BUP) to a European inventory driven by “top-down” information (i.e., TNO_MACC-II; Kuenen et al. 2014; hereinafter referred to as TOD), the reference year being 2009 in both cases. In general, bottom up inventories are defined in terms of administrative divisions whereas EU wide top-down inventories, based on a spatial allocation of country totals, are provided as gridded. For the comparison, the emissions from grid cells belonging to the administrative entity (through comparison with a shape file) are summed up and compared to the regional totals. Note that grid cells crossing the administrative border are fully accounted for. It is important to keep track of this assumption, especially for small administrative regions or/and if the resolution of the top-down inventory is coarse.

The macro-sectors and pollutants considered for the analysis are non-industrial combustion (SNAP02, hereafter referred to as DOM), industrial combustion and processes (SNAP03 + 04, hereafter referred to as INDU), and road transport (SNAP07, hereafter referred to as TRAF). The pollutants considered are NO_*x*_, SO_2_, VOC, and PPM_10_.

For all sectors, NO_*x*_ is selected as the reference pollutant since it is generally accepted that the choice of emission factors and activity shares are similar in the TOD and BUP NO_*x*_ emission inventories. This assumption is in line with the results presented by Granier et al. (2011) in which several global and regional emission inventories were assessed for the 1980–2010 period, the best consensus being found for NO_*x*_ emissions for all periods and all regions. If another pollutant is selected as reference, results will change in the diamond diagram (see point 5 in Screening methodology).

**Fig. 3:**
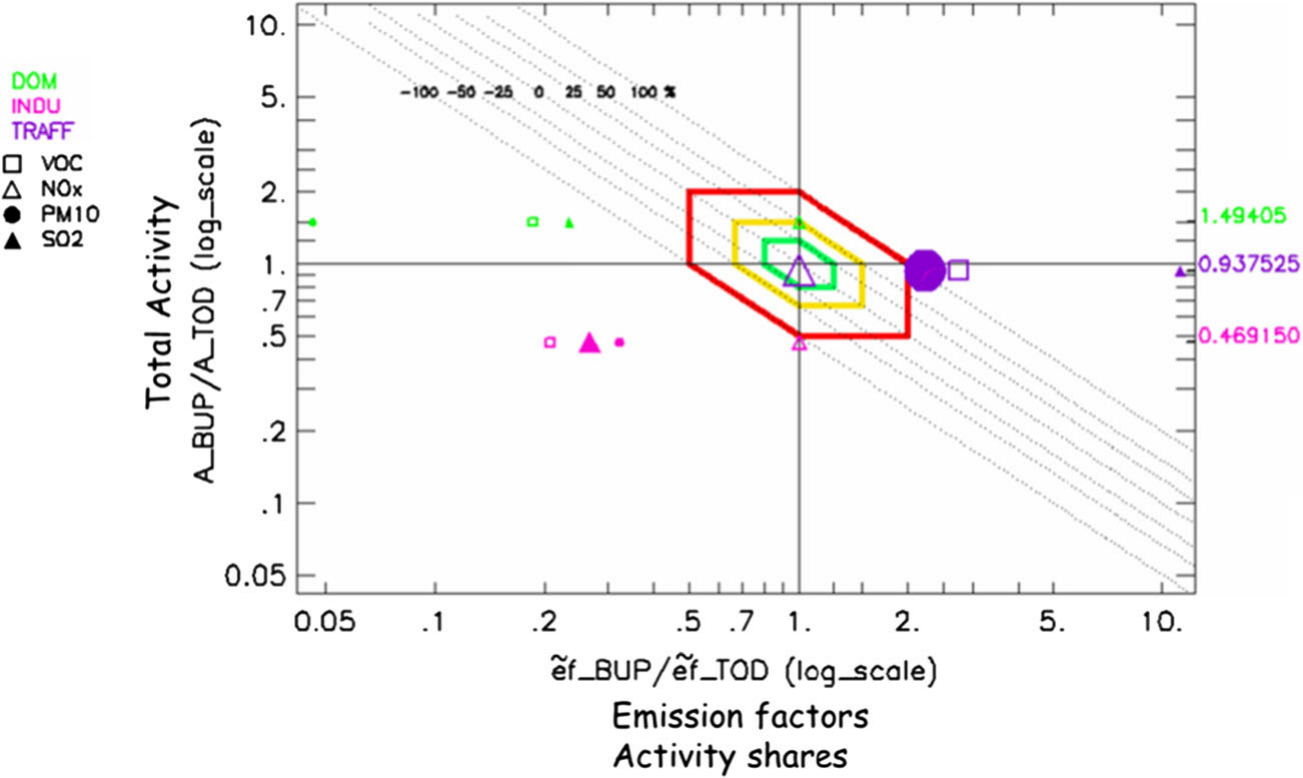
Diamond diagram applied on the test case of Barcelona city for the non-industrial combustion (DOM), industrial combustion and processes (INDU), and road transport (TRAFF) sectors for VOC, NO_*x*_, SO_2_, and PPM_10_

The results for Barcelona are shown in Fig. 3. Moreover, in order to interpret the results and carry out the comparison of the two given inventories, a screening methodology is proposed below.

### Screening methodology

To perform the analysis of Fig. 3, we propose to follow the following approach:

#### Diamond overview

Most of the 12 pollutant-sector comparisons lie outside of the red diamond shape (factor 2), indicating clear issues to be solved.

#### Analysis of total emissions per sector

As mentioned before, all pollutants for one sector lay on the same horizontal line as a result of assumption 2 (i.e., relation 4). In Fig. 3, the bottom-up (BUP) emission estimates for the industrial (INDU) sector clearly underestimate the top-down inventory (TOD) estimate, whereas the contrary is seen for the traffic (TRAF) sector. We identify the over or underestimation of the emissions from the position of the points with respect to the diagonal −1 slope curve. The DOM sector shows both underestimation (PPM_10_, VOC, and SO_2_) and overestimation (NO_*x*_). Because emissions from one specific activity sector should be treated consistently for all the different pollutants in the two inventories, we expect that the points characterizing the different pollutants are very close to each other within a given activity sector. The fact that this is not the case for DOM might point out to issues in terms of emission factors. However, it could also indicate discrepancies in terms of activity shares (i.e., percentage of biomass and natural gas burn) since the difference between biomass-NO_*x*_ emission factors and natural gas-NO_*x*_ emission factors for the DOM sector are usually very low compared to the differences between VOC, PPM_10_-, and SO_*x*_-emission factors (EEA 2013).

The diamond diagram helps also to screen differences in terms of emission magnitude. Indeed, large differences in terms of emission factors, activity, or total emissions between the two inventories might not be a priority if the magnitude of the emissions concerned is small. In Fig. 3, the symbol size for a pollutant is proportional to the quantity of emissions in a given sector compared to the sum of all sectors. This analysis clearly points to VOC and PPM_10_ as main outliers for TRAF and to SO_2_ for the INDU emissions. PPM_10_ for DOM and SO_2_ for TRAF are second priorities. On the other hand, NO_*x*_ for TRAF is the closest to the origin, a reassuring feature on the consistency of the two inventories, considering its size.

#### Emission factors vs. activities

The value of the ratios in the diamond diagram indicates the magnitude of the differences between the two inventories. In the example in Fig. 3, with the exception of a couple of pollutant-sectors (TRAF-NO_*x*_ and DOM-NO_*x*_), all other ratios show larger differences in terms of emission factors than in terms of activities. This is clearly seen by the larger distance from the Y-axis than from the X-axis.

#### Compensation vs. adding up

The DOM points mostly lay in the compensation zone characterized by an overestimation of the activity and an underestimation of the emission factor, whereas for the INDU sector, most of the points lay in the adding-up zone where underestimations in terms of activity and emission factors both contribute to the underestimation of the total emissions. It is interesting to note that some compensation also occurs in terms of sectors. Indeed, while the TRAF sector tends to overestimate all pollutants, the two other sectors tend to underestimate. While the total emissions might be similar, its repartition across different sectors might lead to important impacts, for example in terms of modelled concentrations due to a different spatial allocation of emission per sector in the air quality model. This is particularly relevant in the later assessment of potential measures to improve AQ where an incorrect source representation might lead to budget being allocated to address the wrong sources.

#### Changes in reference pollutant

The choice of the pollutant of reference influences the interpretation of the diagram. Therefore, it is interesting to assess how the diagram changes if another pollutant would be selected as reference. It is important to note that all points in the diagram would only move along their −1 diagonal. These diagonals indicate the total emissions ratios, and these ratios remain unchanged regardless of the choice made for the reference pollutant. If SO_2_ is selected as reference for the TRAF sector, its point would therefore move along the −1 diagonal until it reaches the e/e = 1 line (vertical axis) to satisfy assumption 1 (i.e., relation 3) and therefore stay at coordinates (1, 10). The NO_*x*_ point would also move along its diagonal until it reaches the same horizontal line as SO_2_, i.e., (0.1, 10), and similarly for all other pollutants within this macro-sector. In its new position, NO_*x*_ would be characterized by an underestimation by a factor of 10 of the emission factor, compensated by an overestimation of the activities by a factor 10. Being very improbable, we conclude that SO_2_ cannot be used as reference. If we conduct the same analysis for the INDU and DOM sectors (i.e., selecting SO_2_ as the reference pollutant), the NO_*x*_ point would reach the coordinates (4.5,0.1) and (4.2, 0.35) respectively. In both cases, NO_*x*_ would be characterized by a large underestimation factor of the activity (i.e., up to 10 for the DOM sector) partially compensated by an overestimation of the weighted emissions factors, the results being again very improbable.

A similar reasoning has been followed for other pollutants and sectors to increase our confidence in the choice of our reference pollutant and about the robustness of the analysis.

#### Distances between points

Distances along the horizontal axis provide information on pollutant ratios. A large distance along the horizontal axis between two points indicates issues in terms of emission factors. This is the case for example between NO_*x*_ and SO_2_ for the TRAF sector or between NO_*x*_ and PPM_10_ for the DOM sector. However, this distance also provides information about pollutant ratios and about their consistency between inventories. Indeed, for TRAF the vicinity of the VOC and PPM_10_ along the X-axis implies that:

$$ \frac{{\tilde{e}}_1^{\mathrm{TRAF},\mathrm{V}\mathrm{O}\mathrm{C}}}{{\tilde{e}}_2^{\mathrm{TRAF},\mathrm{V}\mathrm{O}\mathrm{C}}}\approx \frac{{\tilde{e}}_1^{{\mathrm{TRAF},\mathrm{P}\mathrm{P}\mathrm{M}}_{10}}}{{\tilde{e}}_2^{{\mathrm{TRAF},\mathrm{P}\mathrm{P}\mathrm{M}}_{10}}}\iff \frac{{\tilde{e}}_1^{\mathrm{TRAF},\mathrm{V}\mathrm{O}\mathrm{C}}}{{\tilde{e}}_1^{{\mathrm{TRAF},\mathrm{P}\mathrm{P}\mathrm{M}}_{10}}}\approx \frac{{\tilde{e}}_2^{\mathrm{TRAF},\mathrm{V}\mathrm{O}\mathrm{C}}}{{\tilde{e}}_2^{{\mathrm{TRAF},\mathrm{P}\mathrm{P}\mathrm{M}}_{10}}} $$

We deduce therefore that the proportional emission ratio VOC/PPM_10_ for TRAF is consistent in the two inventories, but that both VOC and PPM_10_ are probably overestimated in the BUP inventory, as indicated by the position of these two points with respect to the diagonal. Similarly, we conclude that proportions or pollutant ratios between the VOC, PPM_10_, and SO_2_ emissions (i.e., VOC/PPM_10_, VOC/SO_2_, and PPM_10_/SO_2_) are consistent between the two inventories for INDU, but all pollutants are underestimated with respect to NO_*x*_. For DOM, this consistency is only observed between the VOC and SO2 emissions (i.e., VOC/SO_2_) all pollutants being also underestimated with respect to NO_*x*_.

A similar reasoning can be followed along the Y-axis where the proximity of the three sector lines indicates that the relative sectorial emission ratios are consistent between the two inventories, or in other words:

$$ \frac{A_1^{\mathrm{tot},\mathrm{TRAF}}}{A_2^{\mathrm{tot},\mathrm{TRAF}}}\approx \frac{A_1^{\mathrm{tot},\mathrm{D}\mathrm{O}\mathrm{M}}}{A_2^{\mathrm{tot},\mathrm{D}\mathrm{O}\mathrm{M}}}\iff \frac{A_1^{\mathrm{tot},\mathrm{TRAF}}}{A_1^{\mathrm{tot},\mathrm{D}\mathrm{O}\mathrm{M}}}\approx \frac{A_2^{\mathrm{tot},\mathrm{TRAF}}}{A_2^{\mathrm{tot},\mathrm{D}\mathrm{O}\mathrm{M}}}, $$

with possible generalization of this relation to the INDU sector.

In conclusion, the screening evaluation points out to possible inconsistencies between the two inventories in the TRAF sector, especially for the PPM_10_ and VOC in terms of emission factors. It also points out the need for checking the accuracy of the SO_2_ emission factor for the INDU sector and the issue of the PPM_10_ for INDU and SO_2_ for TRAF and PPM10 for DOM, again in terms of weighted emission factors.

As mentioned earlier, the analysis presented here at macro-sector level should be seen as a first step in the screening process to identify the sectors/pollutant discrepancies. A more detailed approach in terms of sectors is then necessary to identify the causes behind these differences. This is however not the purpose of this work.

## Conclusions

In this work, a methodology has been proposed to compare emission inventories. The main strengths of the approach (differentiating discrepancies in terms of emission factors and activity level) as well as its main limitations (assumption made on the reference pollutant and its influence on the interpretation) have been discussed. A practical example was used to illustrate and explain the methodology, providing relevant information for both emission datasets. The “diamond” diagram is shown to be useful to flag out anomalous behaviors in emission inventories and to get insight in possible explanations. It is particularly helpful to provide information in terms of: discrepancies between total emissions reported by macro-sector and pollutant, contribution of each macro-sector to the total amount of emissions released by pollutant, and identification and quantification of the different factors causing the discrepancies between total emissions. This methodology which must be seen as a first step in an emission inter-comparison exercise, to be completed with other approaches (e.g., GIS or air quality simulation comparison exercise), is general enough to be applied to any type of inventory comparison, independently of the modelling purpose of the inventories. The robustness of the analysis will increase with the level of details (e.g., in terms of sectors). It is therefore advisable to start the analysis with macro-sectors to identify the main differences and pursue it at sector and/or sub-sector level to understand the causes behind these differences.
